# Supplementary material for: A Critical View of the Application of the APEX Software (Aqueous Photochemistry of Environmentally-Occurring Xenobiotics) to Predict Photoreaction Kinetics in Surface Freshwaters
Source: Molecules. 2019 Dec 18;25(1):9. doi: 10.3390/molecules25010009 (PMC7017383; doi:10.3390/molecules25010009)
Supplement: Supplementary file 1 [file molecules-25-00009-s001.zip › 3.2.4_gcc-4.4.0.zip!/3.2.4_gcc-4.4.0/3.2.4_gcc-4.4.0/doc/octave/html/interpreter/Commands-For-Completion.html]

Commands For Completion - Untitled


Next: Commands For History,
Previous: Commands For Text,
Up: Command Line Editing


---

#### 2.4.4 Letting Readline Type For You

The following commands allow Octave to complete command and variable
names for you.

`<TAB>`: Attempt to do completion on the text before the cursor. Octave can complete the names of commands and variables. `M-?`: List the possible completions of the text before the cursor.

— Built-in Function: val = **completion\_append\_char** ()  
— Built-in Function: old\_val = **completion\_append\_char** (new\_val)  
> Query or set the internal character variable that is appended to
> successful command-line completion attempts. The default
> value is `" "` (a single space).

— Built-in Function: **completion\_matches** (hint)  
> Generate possible completions given hint.
>
> This function is provided for the benefit of programs like Emacs which
> might be controlling Octave and handling user input. The current
> command number is not incremented when this function is called. This is
> a feature, not a bug.
